# Carbohydrate Intake and Risk of Cardiovascular Disease: A Systematic Review and Meta-Analysis of Prospective Studies

**DOI:** 10.3390/nu15071740

**Published:** 2023-04-02

**Authors:** Unhui Jo, Kyong Park

**Affiliations:** Department of Food and Nutrition, Yeungnam University, Gyeongsan 38541, Republic of Korea

**Keywords:** carbohydrate, cardiovascular disease, coronary heart disease, stroke, meta-analysis

## Abstract

The purpose of this study is to understand the conflicting results from previous studies on the association between carbohydrate intake and cardiovascular disease (CVD) by conducting a systematic review and meta-analysis to summarize the most recent scientific evidence. A systematic review used three electronic databases to gather literature on the association between carbohydrate intake and CVD. Considering the discrepancies, either fixed or random effect models were chosen to determine the effect size, and sensitivity analysis results, as well as publication bias, were also presented. The meta-analysis found that individuals with the highest carbohydrate intake had a 1.15-fold increased risk of CVD compared to those with the lowest intake (hazard ratio, HR: 1.15, 95% confidence interval, CI: 1.07–1.23). Further subgroup analysis revealed that this association was only present in Asia, with a 1.52-fold increased risk (HR: 1.52, 95% CI: 1.17–1.97), while no associations were seen in the Americas, Europe, and Oceania. The relationship between carbohydrate intake and CVD was non-linear, with a marked escalation beyond 60% of total energy from carbohydrates. Our findings suggest that a high-carbohydrate diet may raise the risk of CVD, particularly in Asian populations. This association may be due to the higher carbohydrate consumption and genetic variations found in Asia.

## 1. Introduction

Cardiovascular disease (CVD) is a major public health challenge worldwide, accounting for a significant proportion of morbidity and mortality, and its prevalence is rapidly increasing, making it a critical global health concern [1]. Unhealthy diets have been identified as a key modifiable risk factor for CVD, including heart disease and stroke. In particular, high-carbohydrate diets have been linked to an increased risk of developing CVD [2]. Studying the impact of macronutrient intake on health outcomes is inherently complex and challenging due to the interdependence of carbohydrates, fats, and proteins. When adjusting one macronutrient’s proportion, the others must also change, assuming stable total energy intake, which complicates isolating the specific effects of each nutrient on health outcomes. The difficulties in assessing macronutrient intake’s impact on health are especially pronounced when examining carbohydrate intake and CVD risk. Numerous studies have explored the relationship between carbohydrate intake and CVD risk, but the diverse definitions of high-carbohydrate intake, inconsistent results, and potential confounding effects of other macronutrients have hindered the establishment of clear conclusions. Nonetheless, studies have quantified dietary carbohydrate intake in various ways, including but not limited to grams per day, total energy-adjusted intake levels, or as a percentage of total calorie intake.

Dietary patterns differ between Western and Asian countries. Western countries typically consume high amounts of protein and fat from sources such as red meat and animal fat, while Asian countries tend to have a diet high in carbohydrates, specifically rice, and low in fat [3,4]. The amount of carbohydrate intake also varies significantly between Asian countries and most European and American countries [5]. This difference in the diet has been shown to have an impact on morbidity and mortality rates [6,7]. Research suggests that blood lipid profiles, which are a risk factor for CVD, are influenced by carbohydrate intake [2,8]. A high-carbohydrate diet may contribute to the development of atherosclerosis and various vascular diseases, ultimately leading to death [9,10]. Therefore, understanding the dietary patterns of different cultures is crucial in developing effective prevention strategies for chronic diseases.

Recently, there has been a growing body of research investigating the association between carbohydrate intake and CVD risk, with some studies suggesting a significant relationship between high-carbohydrate intake and increased CVD risk in Asian populations [11,12,13,14]. However, studies conducted in non-Asian countries have reported mixed results [15,16,17,18], suggesting that the impact of carbohydrate intake on CVD risk may differ depending on the different dietary habits and carbohydrate intake range. These findings highlight the importance of considering cultural and regional dietary patterns in developing effective prevention strategies for chronic diseases, particularly CVD. As such, a systematic review of recent studies on the relationship between carbohydrate intake and CVD, with a specific focus on studies conducted in Asian populations, is warranted.

The aim of this systematic review and meta-analysis is to analyze high-quality prospective cohort studies to establish the temporal sequence between diet assessment and subsequent CVD incidence and to provide a more reliable understanding of the association between carbohydrate intake and the risk of CVD. Specifically, we aim to conduct a meta-analysis to summarize the overall findings and identify any inconsistencies in the results. Additionally, we will focus on recent studies, particularly those conducted in Asian populations, where carbohydrate intake tends to be relatively high compared to Western populations. This approach will provide valuable insights into the complex relationship between carbohydrate intake and CVD risk in different populations and inform the development of more effective prevention strategies for chronic diseases.

## 2. Materials and Methods

### 2.1. Search Strategy

This meta-analysis adhered to the guidelines outlined in the Preferred Reporting Items for Systematic Reviews and Meta-Analyses (PRISMA) [19]. An extensive literature search was performed to identify studies examining the relationship between carbohydrate intake and CVD, with the search limited to studies published up until 13 September 2022.

We used three major electronic databases, PubMed, Scopus, and Web of Science. Our search strategy consisted of three groups of keywords related to dietary carbohydrates, CVD, and prospective cohort studies: Keyword group 1: “dietary carbohydrate”, “carbohydrate”; keyword group 2: “infarction”, “heart arrest”, “stroke”, “cardiovascular”, “heart disease”, “myocardial ischemia”, “coronary artery disease”, “coronary heart disease”, “atherosclerosis”, “hypertension”, ”acute coronary syndrome”, ”coronary bypass surgery”, ”percutaneous revascularization”, ”cerebral infarction”, ”cerebral hemorrhage”; and keyword group 3: “prospective”, “longitudinal”, “cohort”, “cohort studies”. We also manually examined the reference lists of all retrieved articles and searched for conference articles, resulting in the inclusion of one unpublished dataset. Two researchers independently conducted the searches, and any discrepancies were resolved through discussion. Our study selection criteria were based on the “participant, intervention (exposure), comparison, outcome, and study design” framework, which is outlined in Table 1. This rigorous search methodology ensures that we have included all relevant studies on the topic and provides a solid foundation for our systematic review and meta-analysis.

### 2.2. Study Selection

The selection criteria for this systematic review and meta-analysis were designed to identify high-quality prospective cohort studies that examined the relationship between carbohydrate intake and CVD risk in adult populations. Specifically, the criteria required that studies present hazard ratio (HR) or relative risk (RR) with a 95% confidence interval (CI) for CVD or coronary heart disease (CHD) risk in relation to carbohydrate intake. Studies were excluded from the meta-analysis if they were not published in English or Korean, conducted on patients with diabetes mellitus, or presented only glycemic index, glycemic load, or carbohydrate score as dietary information when analyzing the association between carbohydrate intake and CVD outcomes. In addition, articles that were not original, including but not limited to reviews, meta-analyses, letters, and editorials, as well as non-human studies such as animal or in vivo studies, were not considered for inclusion. In the case of multiple articles using the same dataset, only the article with the most recent data or the longest follow-up period was included in order to avoid duplicative or outdated data. The selection process was rigorous and involved a detailed examination of each study to ensure that it met the criteria for inclusion. By including only high-quality studies in the analysis, this systematic review and meta-analysis provide a more comprehensive and reliable understanding of the relationship between carbohydrate intake and CVD risk in different populations.

### 2.3. Data Extraction and Quality Assessment

Two investigators independently evaluated the quality of the selected articles using the Newcastle–Ottawa Scale (NOS) for prospective cohort studies [20]. The NOS consists of eight questions that assess aspects of selection, comparability, and outcome, and each question is scored from 0 to 9 points. All 23 studies were scored and evaluated as good, fair or poor. Of the studies, 21 studies were evaluated as good, and 2 studies were evaluated as fair (as seen in Supplementary Table S1).

### 2.4. Statistical Analysis

A meta-analysis was performed on 23 studies to determine the overall risk estimate of CVD associated with the highest intake of carbohydrates compared to the lowest intake. When a study reported results for the association between carbohydrate intake and different types or definitions of CVD-related diseases, the outcomes were defined according to the original article’s criteria and prioritized in the following order: CVD, CHD, and stroke.

For studies that reported separate results for men and women, data for each gender were analyzed separately. In cases where a study included participants from multiple countries, data from the continent where the majority of the countries belonged were used as a representative sample. The heterogeneity of effect size was evaluated using Higgin’s *I*^2^, which categorized it as low (≤25%), moderate (<25–75%), or high (≥75%) heterogeneity [21]. Both a fixed-effect model, which only takes into account sampling error, and a random-effect model, which also considers variation between studies, were used in this analysis. When significant heterogeneity was identified, a subgroup analysis was conducted. A sensitivity analysis using the leave-one-out method was performed to assess the robustness of the results, which presented the mean effect sizes with each study removed. Additionally, a funnel plot was used to evaluate the potential publication bias from small sample studies on the results.

A two-stage dose–response random-effects meta-regression was performed to pool the risk estimates of CVD according to carbohydrate intake from each study. The linear trend of the association between carbohydrate intake and CVD risk was tested using the Wald test, and a non-linear relationship was examined using a restricted cubic spline with carbohydrate intake ranges divided using four knots (5%, 35%, 65%, and 95%) [22,23]. This method could only be used when the risk for CVD was presented in HR or RR with its CI for at least three quantitative exposure categories of carbohydrate intake. Studies that did not present carbohydrate intake range or used different category units were excluded. As a result, a total of eight studies were included in the dose–response meta-analysis. The meta-analysis was performed using the STATA version 13.1 (Stata Corp., College Station, TX, USA) software.

## 3. Results

### 3.1. Literature Search

Supplementary Figure S1 provides a flow chart depicting the process of identifying the articles included in this meta-analysis. The search was conducted across three electronic databases, PubMed, Scopus, and Web of Science, resulting in a total of 3463 articles. Following a screening process, 1038 duplicate articles were excluded, and 2377 articles that did not meet the inclusion criteria were dropped after a review of their title and abstract. This resulted in a total of 49 articles that were subjected to a more detailed review of the full text. Of these, 26 studies were excluded as they did not meet specific inclusion criteria, such as not reporting total carbohydrate intake, only using the glycemic index, glycemic load, or carbohydrate score, not presenting HR/RR, being cross-sectional studies, or analyzing duplicate datasets. Finally, a total of 23 articles [6,12,13,14,15,16,17,18,24,25,26,27,28,29,30,31,32,33,34,35,36,37,38] were included in the meta-analysis. Overall, Supplementary Figure S1 provides a comprehensive overview of the study selection process, including the specific inclusion and exclusion criteria.

All the participants of these studies were aged 18 and over, and the studies were conducted across various continents, including the Americas, Europe, Oceania, and Asia (Table 2).

### 3.2. Pooled Results on the Association between Carbohydrate Intake and CVD

**Figure 1:**
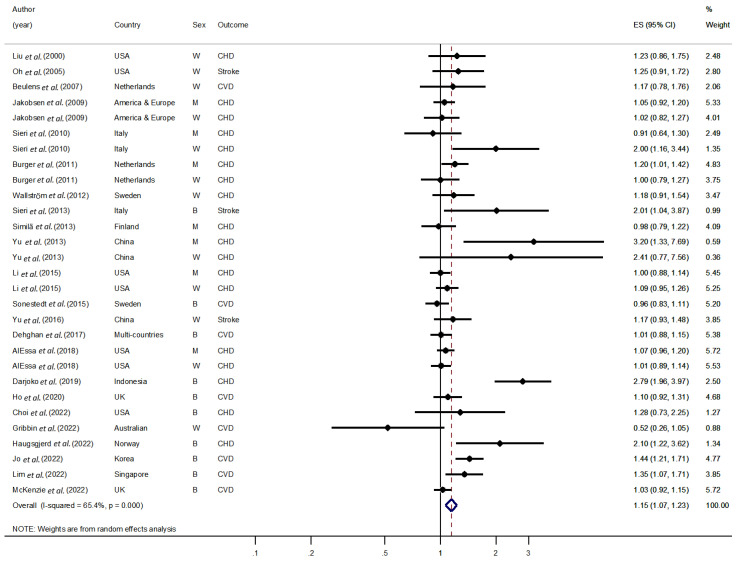
Forest plot of the association between carbohydrate intake and cardiovascular disease. ES, effect size; CVD, cardiovascular disease; CHD, coronary heart disease; W, women; M, men; USA, United States of America; UK, United Kingdom [6,12,13,14,15,16,17,18,25,26,27,28,29,30,31,32,33,34,35,36,37,38,39].

Figure 1 illustrates the association between carbohydrate intake and the risk of total CVD, as primarily reported in the original articles for CVD, CHD, or stroke. The majority of the studies included in this analysis are located on the right side of the plot, with a RR or HR around 1, indicating a positive or null association between carbohydrate intake and CVD risk. However, one study by Gribbin *et al*. that focused on women and CVD was located on the left side, indicating a non-significant result. When all the results are combined, they suggest that increasing carbohydrate intake is associated with a higher risk of total CVD (HR/RR: 1.15, 95% CI: 1.07–1.23) and that there is moderate heterogeneity among the studies (*I*^2^ = 65.4%).

**Figure 2:**
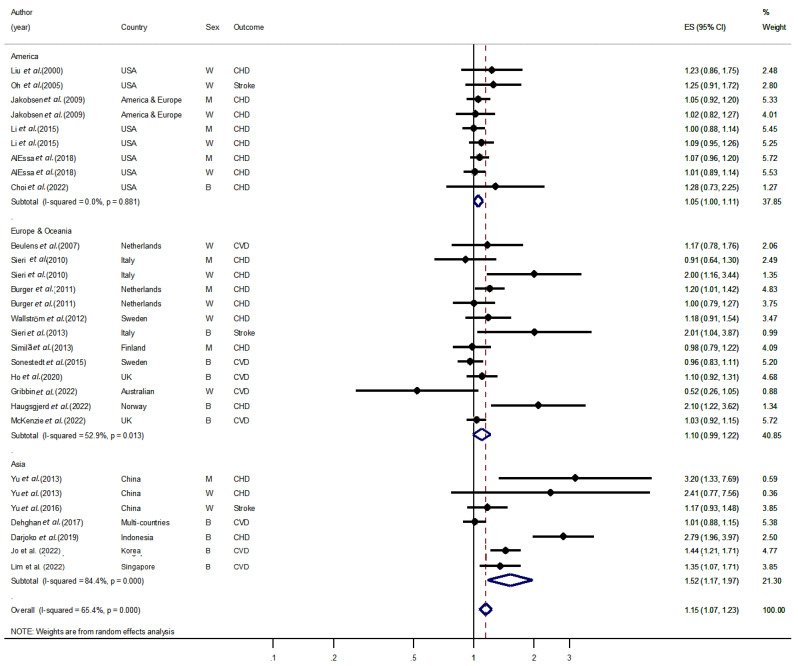
Forest plot of the association between carbohydrate intake and cardiovascular disease stratified by continent. ES, effect size; CVD, cardiovascular disease; CHD, coronary heart disease; W, women; M, men; USA, United States of America; UK, United Kingdom [6,12,13,14,15,16,17,18,25,26,27,28,29,30,31,32,33,34,35,36,37,38,39].

The subgroup analyses conducted in this study to evaluate the impact of geographic location on the relationship between carbohydrate intake and CVD risk provide important insights (Figure 2). The findings suggest that there is no significant association between carbohydrate intake and CVD risk in the Americas, Europe, and Oceania. However, the pooled results from Asian countries showed that higher carbohydrate intake is associated with a significant increase in CVD risk, with a 52% increase in risk (*I*^2^ = 84.4%, HR/RR: 1.52, 95% CI: 1.17–1.97). These findings suggest that the impact of carbohydrate intake on CVD risk may be influenced by geographic location.

**Figure 3:**
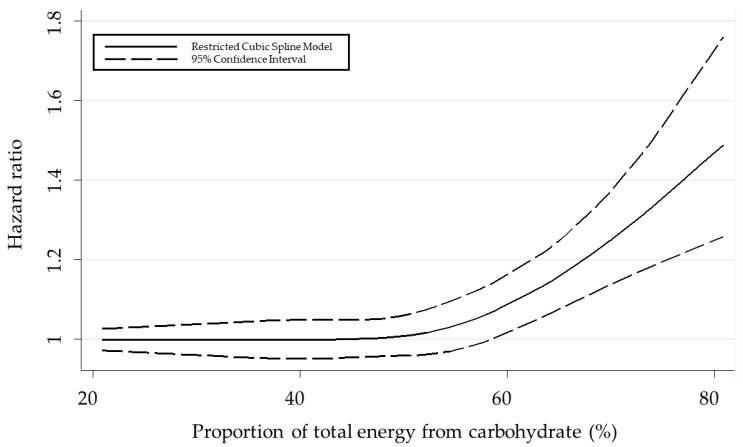
Dose–response analysis between carbohydrate intake and cardiovascular disease.

To better understand the dose–response relationship between carbohydrate intake and CVD risk, a restricted cubic splines model was employed in this study. The analysis presented in Figure 3 revealed a non-linear trend in the relationship, with CVD risk increasing gradually up to 60% of total energy from carbohydrate intake. Beyond this point, the risk increased dramatically, suggesting that there may be a threshold beyond which the impact of carbohydrate intake on CVD risk becomes particularly pronounced. The relationship was found to be non-linear, with a significant inflection point at 60% of total energy from carbohydrate intake (*p* for non-linearity = 0.0002).

### 3.3. Influence Analysis and Publication Bias

The sensitivity analysis conducted in this study provides important information about the robustness of the overall findings. Supplementary Figure S2 illustrates the results of the sensitivity analysis, which showed that the result of the Darjoko *et al*. study appeared to be somewhat distant from the other studies, with an overall mean of 1.15. However, this outlier did not substantially impact the overall result of the meta-analysis. In addition, the study employed a funnel plot and trim and fill analysis to test for publication bias, as shown in Supplementary Figures S3 and S4, respectively. Figure S3 revealed an asymmetric funnel plot, and the trim and fill analysis determined that an additional six studies were needed to balance the asymmetry. After accounting for these extra studies, the effect size of the adjusted values slightly decreased compared to the initial results; however, the association remained consistent. This consistency in the adjusted findings further strengthens the overall robustness of our study.

## 4. Discussion

This meta-analysis of prospective cohort studies suggests that a higher intake of carbohydrates is independently associated with an increased risk of CVD, particularly in Asian populations. Compared to those in the lowest category of carbohydrate intake, individuals in the highest category had a significantly greater risk of CVD (52%) in Asia. However, the risk was minimal in the Americas, Oceania, and Europe, and these differences were not statistically significant. A dose–response meta-analysis revealed that the risk of CVD increases dramatically in a non-linear manner when carbohydrate intake exceeds 60%. This finding has important implications for clinical practice as it highlights the potential impact of a carbohydrate-based diet on CVD risk, particularly in Asian populations and could be used as a strategy for reducing CVD.

Our research findings have shed light on an intriguing observation regarding the relationship between carbohydrate consumption and CVD risk, with the association appearing to be more pronounced in Asian populations. While it is well-known that Asian populations tend to consume a diet that is high in carbohydrates [39,40,41,42], our study has highlighted how this may be contributing to their increased risk of CVD. Furthermore, we speculate that this increased risk may be due to a combination of higher carbohydrate intake levels and genetic variations within these populations. For instance, a prior study found that the proportion of total energy derived from carbohydrates was 81.5% in Korea and 65.75% in the US. Additionally, a significant portion of the Korean population (two-thirds) exceeded the acceptable macronutrient distribution range, while only 8% of the American population did the same, highlighting a notable difference in carbohydrate intake levels between the two countries [7]. This, in combination with genetic variations, could be contributing to the increased risk of CVD. For example, research has found that East Asians have a reduced innate ability to secrete insulin, which leads to decreased capacity with aging or accelerated β-cell exhaustion compared to other ethnicities [43,44]. This ethnic difference in genetic susceptibility may influence the relationship between carbohydrate intake and CVD risk differently. Among the Asian population, even a small decline in insulin secretory function can result in a rapid decrease in the threshold level of insulin resistance and type 2 diabetes, which are strong risk factors for CVD [43,44].

The effect of high-carbohydrate intake on health outcomes has been extensively studied in various populations, and recent evidence suggests that the harmful health consequences may be more pronounced in Asian populations [6,7]. For instance, a study of the Prospective Urban Rural Epidemiology cohort in 18 countries across five continents found that all-cause mortality was significantly higher among Asian populations with increasing carbohydrate intake [6]. Given these findings, the Korean Society of Lipidology and Atherosclerosis has emphasized the importance of establishing dietary guidelines to limit carbohydrate intake in Korea, as a high daily intake of carbohydrates is typical among Koreans [45]. The Korean Society of Lipidology and Atherosclerosis explained that Western populations generally consume fewer carbohydrates in their diet, and thus, no dietary recommendations have been published for restricting carbohydrate intake in these populations [45]. These findings suggest that there may be significant ethnic and cultural differences in carbohydrate metabolism and health outcomes, which highlights the importance of personalized interventions to reduce the risk of chronic diseases.

The effect of excessive carbohydrate consumption on human health is complex and can result in adverse effects on the liver’s ability to convert carbohydrates into fat and inhibit the synthesis of beneficial high-density lipoprotein cholesterol (HDL-C) [8,46,47,48,49]. This disruption can lead to elevated levels of triglycerides and reduced levels of HDL-C, both of which are major risk factors for metabolic disorders such as insulin resistance and metabolic syndrome [50]. These conditions are considered to be major predictors of a high 10-year risk for CVD by the World Health Organization [2]. Furthermore, in cultures that traditionally consume high-carbohydrate diets, there may be a lower consumption of other essential nutrients, such as protein and fats, resulting in a nutrient imbalance. This imbalance can significantly impact overall health and increase the risk of developing CVD [51,52,53,54]. In order to mitigate these risks, it is essential to promote a balanced diet that incorporates adequate intake ratios of carbohydrates, protein, and fats. A balanced intake of macronutrients can help to ensure the body has the necessary components to function optimally, regulate blood sugar levels, promote healthy cholesterol levels, and reduce the risk of chronic diseases, including CVD.

This study presents some intriguing findings, but there are a few limitations to consider. Firstly, the studies in the meta-analysis endeavored to control for potential confounding factors, but residual confounding may still be present. For instance, although all studies adjusted for total calorie intake, a noticeable disparity existed in the adjustment for fat intake. Considering that fat intake is an established risk factor for CVD and frequently correlates with variations in carbohydrate intake, this inconsistency might have impacted the conclusions of our meta-analysis. Moreover, the inconsistent adjustment for covarying dietary macronutrients may confound and potentially bias our results, as different macronutrients can interact with each other and have varying effects on health outcomes. Future studies should account for these interactions to provide more accurate and robust conclusions. Secondly, variations in how dietary surveys were conducted among the studies could have influenced the results. Thirdly, this study determined carbohydrate intake levels by using the proportion of total energy from carbohydrate intake based solely on calorie intake, without considering the types or quality of carbohydrates. Lastly, this meta-analysis was not preregistered on the “International Prospective Register of Systematic Reviews“, which may limit the transparency and reproducibility of our methods and results. Despite these limitations, this study has several strengths. To the best of our knowledge, this is the first systematic review and meta-analysis that summarizes the current literature on the relationship between carbohydrate intake levels and CVD, taking into consideration dietary habits by culture or geographical region. This is a significant contribution to the existing research on this topic, and the findings could be utilized to inform future research and public health interventions that consider the impact of culture and geography on dietary habits. The study’s methodology, including the search strategy and inclusion criteria, was robust and comprehensive, enhancing the study’s validity. Additionally, the study’s reliability is enhanced by the use of prospective cohort studies in the meta-analysis, which provide stronger evidence of causality compared to case-control or cross-sectional designs. Overall, the study’s strengths outweigh its limitations and highlight the need for further research to establish clear dietary guidelines that could reduce CVD risk across different populations while also taking into account cultural and geographical differences in dietary habits.

## 5. Conclusions

In conclusion, the meta-analysis of 23 cohort studies found that high-carbohydrate intake, particularly over 60% of total energy from carbohydrates, can have adverse effects on the incidence of CVD, with a stronger association in Asians with a carbohydrate-based diet and weaker insulin sensitivity. These findings have significant implications for the ongoing efforts to reduce the incidence of CVD by providing ethnic-specific scientific evidence for prevention. However, further research is required to better understand the relationship between carbohydrate intake and CVD risk in Asian populations. It is essential to investigate the effects of high-carbohydrate diets in prospective cohort studies involving various age groups, along with a detailed analysis of types and food sources of carbohydrates. Such studies could provide a more comprehensive understanding of the mechanisms by which carbohydrate intake influences CVD risk and the potential benefits of specific dietary interventions. With this knowledge, public health interventions can be tailored to reduce the incidence of CVD and promote healthy dietary habits across diverse populations, thus enhancing global efforts to combat this significant public health challenge.

## Figures and Tables

**Table 1:** PICOS criteria for inclusion and exclusion of studies.

Population	Adults
Exposure	Carbohydrate intake
Comparator	Highest vs. lowest categories of exposure
	Reference range vs. every range of exposure
Outcomes	Cardiovascular disease
Study design	Cohort study
PICOS, participant, intervention (exposure), comparison, outcome, and study design	

**Table 2:** Characteristics of studies included in the meta-analysis.

Author (Year)	Country	Age (Year)	N	Sex	Study Name	Follow-Up Year	Dietary Assessment Method	Outcomes	HR/RR (95% CI)	Adjustment	Study Quality Score
Liu *et al.* (2000) [24]	USA	38–63	75,521	W	NHS	10	FFQ	CHD	1.23 (0.86–1.75)	Residual energy-adjusted carbohydrate, age, BMI, cigarette smoking, alcohol intake, parental family history of myocardial infarction before the age of 60 y, self-reported history of hypertension or history of high cholesterol, menopausal status, aspirin use, use of multiple vitamin or vitamin E supplement, physical activity, protein intake, intake of saturated, polyunsaturated, trans fats, dietary fiber, vitamin E, folate, and total energy intake	7
Oh *et al.* (2005) [25]	USA	30–55	78,779	W	NHS	18	FFQ	Stoke	1.25 (0.91–1.73)	Age, BMI, smoking, alcohol intake, parental history of MI, history of hypertension, hypercholesterolemia, diabetes, menopausal status, postmenopausal hormone use, aspirin use, multivitamin use, vitamin E supplement use, physical activity, energy, cereal fiber, saturated fat, MUFA, PUFA, trans fat, and omega-3 fatty acids	7
Beulens *et al.* (2007) [26]	Netherlands	49–70	15,714	W	EPIC	9	FFQ	CVD	1.17 (0.78–1.77)	Age, hypertension, cholesterolemia, smoking, BMI, mean systolic blood pressure, total physical activity, menopausal status, hormone replacement therapy use, oral contraceptives use, alcohol intake, total energy intake, energy-adjusted intake of vitamin E, protein, dietary fiber, folate, saturated fat, MUFA, and PUFA	6
Jakobsen *et al.* (2009) [27]	American and Europe	47–61	344,696	M W	AHS, ARIC, ATBC, FMC, GPS, HPFS, IIHD, IWHS, NHS, VIP, WHS	4–10	FFQ or DH	CHD	1.05 (0.92–1.21) 1.02 (0.82–1.28)	Intakes MUFA, PUFA, trans fat, protein, glycemic carbohydrate, energy, smoking, BMI, physical activity, education, alcohol intake and history of hypertension	5
Sieri *et al.* (2010) [28]	Italy	M: 35–64 W: 35–74	47,749	M W	EPICOR	7.9	FFQ	CHD	0.91 (0.64–1.30) 2.00 (1.16–3.43)	Non-alcohol energy intake, hypertension, smoking, education, categories of alcohol intake, BMI, fiber intake, and physical activity	7
Burger *et al.* (2011) [29]	Netherlands	21–64	19,608	M W	EPIC-MORGEN	11.9	FFQ	CHD	1.20 (1.02–1.43) 1.00 (0.79–1.28)	Age, smoking packyears, education, BMI, physical activity, hypertension, oral contraceptive use, total energy, energy-adjusted nutrients (alcohol, vitamin C, dietary fiber, saturated fat, monounsaturated fat), plasma total cholesterol, and HDL-C	6
Wallström *et al.* (2012) [30]	Sweden	44–73	20,674	W	MDCS	13.5 (mean)	FFQ & 7-days DH	CHD	1.18 (0.91–1.54)	Age, method version, total energy intake, season, BMI class, smoking category, education, alcohol category, systolic blood pressure, antihypertensive treatment, antihyperlipidemic treatment, leisure time physical activity, and quintiles of energy-adjusted dietary fiber	7
Sieri *et al.* (2013) [31]	Italy	35–75	44,099	B	EPIC	10.9	FFQ	Stoke	2.01 (1.04–3.86)	Sex, age, education, smoking, BMI, alcohol, non-alcohol, energy intake, cereal fiber intake, saturated fat, monounsaturated fat, polyunsaturated fat, and physical activity	7
Similä *et al.* (2013) [32]	Finland	50–69	21,955	M	ATBC	19	FFQ	CHD	0.98 (0.79–1.22)	Age, intervention group, smoking, BMI, physical activity, serum total and HDL-C, blood pressure, and intakes of energy, alcohol, total fat, protein, magnesium, and potassium	6
Yu *et al.* (2013) [33]	China	M: 40–74 W: 40–70	117,366	M W	SWHS & SMHS	5.4 (mean)	FFQ	CHD	3.20 (1.33–7.68) 2.41 (0.77–7.57)	Age, educational level, income, smoking status, alcohol consumption, physical activity level, waist-to-hip ratio, history of hypertension, dietary intakes of total energy, saturated fat, and protein	7
Li *et al.* (2015) [34]	USA	M: 40–75 W: 30–55	127,536	M W	NHS & HPFS	M: >24 W: >30	FFQ	CHD	1.00 (0.88–1.14) 1.09 (0.94–1.25)	Total energy intake, the energy contribution from protein, cholesterol intake, alcohol intake, smoking status, BMI, physical activity, use of vitamins, aspirin, family history of MI, diabetes, presence of baseline hypercholesterolemia and hypertension, and percentage of energy from carbohydrates from whole grains, from refined starches/sugars and from other foods simultaneously	7
Sonestedt *et al.* (2015) [35]	Sweden	44–74	26,445	B	MDCS	14	DH	CVD	0.96 (0.83–1.11)	Age, sex, season, diet method version, energy intake, BMI, smoking, alcohol consumption, leisure-time physical activity, and education	7
Yu *et al.* (2016) [36]	China	40–70	64,328	W	SWHS	10 (mean)	FFQ	Stoke	1.17 (0.92–1.47)	Age, education, cigarette smoking, BMI, family history of stroke, history of hypertension, history of dyslipidemia, total energy intake, saturated fat intake, and a partial diet quality score	7
Dehghan *et al*. (2017) [6]	Multicenteris	35–70	135,335	B	PURE study	7.4	FFQ	CVD	1.01 (0.88–1.15)	Age, sex, education, waist-to-hip ratio, smoking, physical activity, diabetes, urban or rural location, and energy intake	6
AlEssa *et al.* (2018) [37]	USA	M: 40–75 W: 30–55	117,885	M W	NHS & HPFS	M: 26 F: 28	FFQ	CHD	1.07 (0.96–1.20) 1.01 (0.89–1.14)	Age, BMI, family history of CHD, smoking status, alcohol intake, physical activity level, multivitamin use, aspirin use, vitamin E use, race, total energy, polyunsaturated fat–to–saturated fat ratio and trans fat	7
Darjoko *et al.* (2019) [12]	Indonesia	Over 25	4840	B	CS-RFNCD	6	FFQ & food recall questionnaire	CHD	2.79 (1.96–3.97)	-	5
Ho *et al.* (2020) [38]	UK	37–73	195,658	B	UK Biobank	10.6	24 h RC	CVD	1.10 (0.91–1.30)	Age, sex, deprivation index, ethnicity, smoking status, height, BMI, systolic blood pressure, baseline diabetes, mental health disorders, total physical activity, daily alcohol intake, and total energy intake	7
Choi *et al.* (2022) [15]	USA	18–30	4701	B	CARDIA	32 (median)	Interviewer-administered DH	CHD	1.28 (0.72–2.22)	Age at baseline, sex, race, total energy intake, maximal educational attainment, parental history of CVD, pack-years of smoking, physical activity level, use of lipid-lowering medications, and BMI	6
Gribbin *et al.* (2022) [16]	Australia	52.5 ± 1.5	9899	W	ALSWH	15	FFQ	CVD	0.52 (0.26–1.06)	Age, menopausal status, country of birth, area of residence, occupation, education, household income, marital status, smoking status, physical activity levels, BMI, polycystic ovary syndrome, gestational diabetes mellitus, hypertension, diabetes mellitus, % saturated fat intake, % PUFA, % MUFA, % cholesterol, % alcohol, fiber, glycemic index, and glycemic load	6
Haugsgjerd *et al*. (2022) [17]	Norway	46–49	2995	B	HUSK	10.8 (mean)	FFQ	CHD	2.10 (1.22–3.63)	Age, sex, energy intake, physical activity, and smoking	7
Jo *et al.* (2022) [13]	Korea	over 40	173,696	B	KoGES	KARE: 9.59 HEXA: 4.25	FFQ	CVD	1.44 (1.21–1.71)	Age, sex, household income, smoking status, alcohol consumption status, physical activity level, and obesity status	6
Lim *et al.* (2022) [14]	Singapore	21–65	12,408	B	Singapore MEC	10.1 (mean)	FFQ	CVD	1.35 (1.07–1.71)	Age, sex, ethnicity, total energy intake, moderate-to-vigorous physical activity, smoking, alcohol consumption, educational level, history of diabetes, hypertension, dyslipidemia, family history of heart disease, menopausal status, BMI, intake of fiber and cholesterol for carbohydrate, and mutually adjusted for intake of all other nutrients except for carbohydrate for the remaining nutrients	7
McKenzie *et al.* (2022) [18]	UK	40–69	120,963	B	UK biobank	11.1 (mean)	Two or more 24 h RC	CVD	1.03 (0.92–1.15)	Age, smoking, sex, height, weight, mean alcohol intake, physical activity, systolic blood pressure, Townsend score, diabetes, lipid-lowering medication, and antihypertensive medication	7

HR: hazard ratio, RR: relative risk, CI: confidence interval, M: men, W: women, CVD: cardiovascular disease, CHD: coronary heart disease, MI: myocardial infarction, USA: united states of America, UK: united kingdom, FFQ: food frequency questionnaire, DH: dietary history, 24h RC: a 24-h dietary recall, NHS: The Nurses’ Health Study, EPIC: European Prospective Investigation into Cancer and Nutrition, AHS: Adventist Health Study, ARIC: Atherosclerosis Risk in Communities Study, ATBC: Alpha-Tocopherol and Beta-Carotene Cancer Prevention Study, FMC: Finnish Mobile Clinic Health Study, GPS: Glostrup Population Study, HPFS: The Health Professionals Follow-Up Study, IIHD: Israeli Ischemic Heart Disease Study, IWHS: Iowa Women’s Health Study, VIP: Vasterbotten Intervention Program, WHS: Women’s Health Study, EPICOR: large EPIC-Italy, MDCS: Malmo Diet and Cancer Study, SWHS: Shanghai Women’s Health Study, SMHS, Shanghai Men’s Health Study, PURE: The Prospective Urban Rural Epidemiology, CS-RFNCD: cohort study on risk factors of non-communicable diseases, HUSK: Hordaland Health Study, ALSWH: Australian Longitudinal Study on Women’s Health, CARDIA: Coronary Artery Risk Development in Young Adults, MEC: Multi-Ethnic Cohort, KoGES, Korean Genome and Epidemiology Study, KARE, Korea Association Resource, HEXA, Health Examinee. BMI: body mass index, MUFA: monounsaturated fatty acids, PUFA: polyunsaturated fatty acids, HDL-C: high-density lipoprotein cholesterol.

## Data Availability

Not applicable.

## Supplementary Materials

The following supporting information can be downloaded at: https://www.mdpi.com/article/10.3390/nu15071740/s1, Table S1: Quality assessment of included studies by NOS for prospective cohort studies; Figure S1: Flow chart of study selection for the meta-analysis; Figure S2: Influence analysis of pooled hazard ratios; Figure S3: Funnel plot for publication bias; Figure S4: Trim and fill.
