# Supplementary material for: Carbohydrate Intake and Risk of Cardiovascular Disease: A Systematic Review and Meta-Analysis of Prospective Studies
Source: Nutrients. 2023 Apr 2;15(7):1740. doi: 10.3390/nu15071740 (PMC10096555; doi:10.3390/nu15071740)
Supplement: Supplementary file 1 [file nutrients-15-01740-s001.zip › nutrients-2263258-supplementary.pdf]

Supplementary Material

Article Title

Carbohydrate intake and risk of cardiovascular disease: A systematic review and meta-analysis of prospective studies

Unhui Jo and Kyong Park*

*** Correspondence:** Kyong Park: kypark@ynu.ac.kr

Table S1. Quality assessment of included studies by NOS for prospective cohort studies

No.	Study (year)	Score									
		Selection			Comparability		Outcome			Total	Quality
		1	2	3	4	5	6	7	8		
1	Liu *et al*. (2000)	1	1	0	1	1	1	1	1	7	Good
2	Oh *et al*. (2005)	1	1	0	1	1	1	1	1	7	Good
3	Beulens *et al*. (2007)	1	1	0	1	1	1	1	0	6	Good
4	Jakobsen *et al*. (2009)	1	1	0	1	1	0	1	0	5	Fair
5	Sieri *et al*. (2010)	1	1	0	1	1	1	1	1	7	Good
6	Burger *et al*. (2011)	1	1	0	1	1	1	1	0	6	Good
7	Wallström *et al*. (2012)	1	1	0	1	1	1	1	1	7	Good
8	Sieri *et al*. (2013)	1	1	0	1	1	1	1	1	7	Good
9	Similä *et al*. (2013)	1	1	0	1	1	1	1	0	6	Good
10	Yu *et al*. (2013)	1	1	0	1	1	1	1	1	7	Good
11	Li *et al*. (2015)	1	1	0	1	1	1	1	1	7	Good
12	Sonestedt *et al*. (2015)	1	1	0	1	1	1	1	1	7	Good

13	Yu *et al*. (2016)	1	1	0	1	1	1	1	1	7	Good
14	Dehghan *et al*. (2017)	1	1	0	1	1	0	1	1	6	Good
15	AlEssa *et al*. (2018)	1	1	0	1	1	1	1	1	7	Good
16	Darjoko *et al*. (2019)	1	1	0	1	0	1	1	0	5	Fair
17	Ho *et al*. (2020)	1	1	1	1	1	1	1	0	7	Good
18	Choi *et al*. (2022)	1	1	1	0	1	1	1	0	6	Good
19	Gribbin *et al*. (2022)	1	1	0	1	2	0	1	0	6	Good
20	Haugsgjerd *et al*. (2022)	1	1	0	1	1	1	1	1	7	Good
21	Jo *et al*. (2022)	1	1	1	1	1	0	1	0	6	Good
22	Lim *et al*. (2022)	1	1	0	1	1	1	1	1	7	Good
23	McKenzie *et al*. (2022)	1	1	1	1	1	1	1	0	7	Good

NOS, Newcastle–Ottawa Scale.

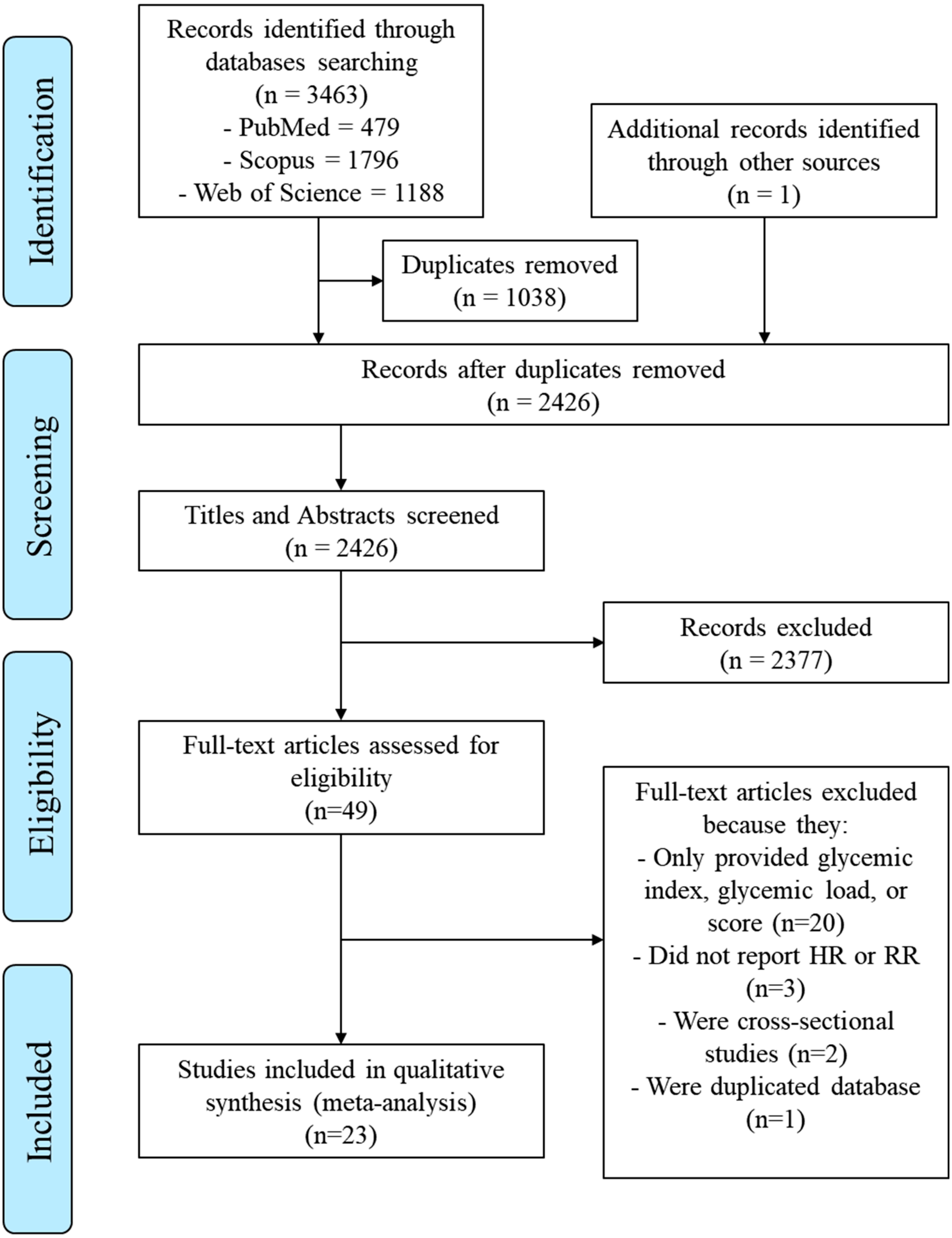

Figure S1. Flow chart of study selection for the meta-analysis

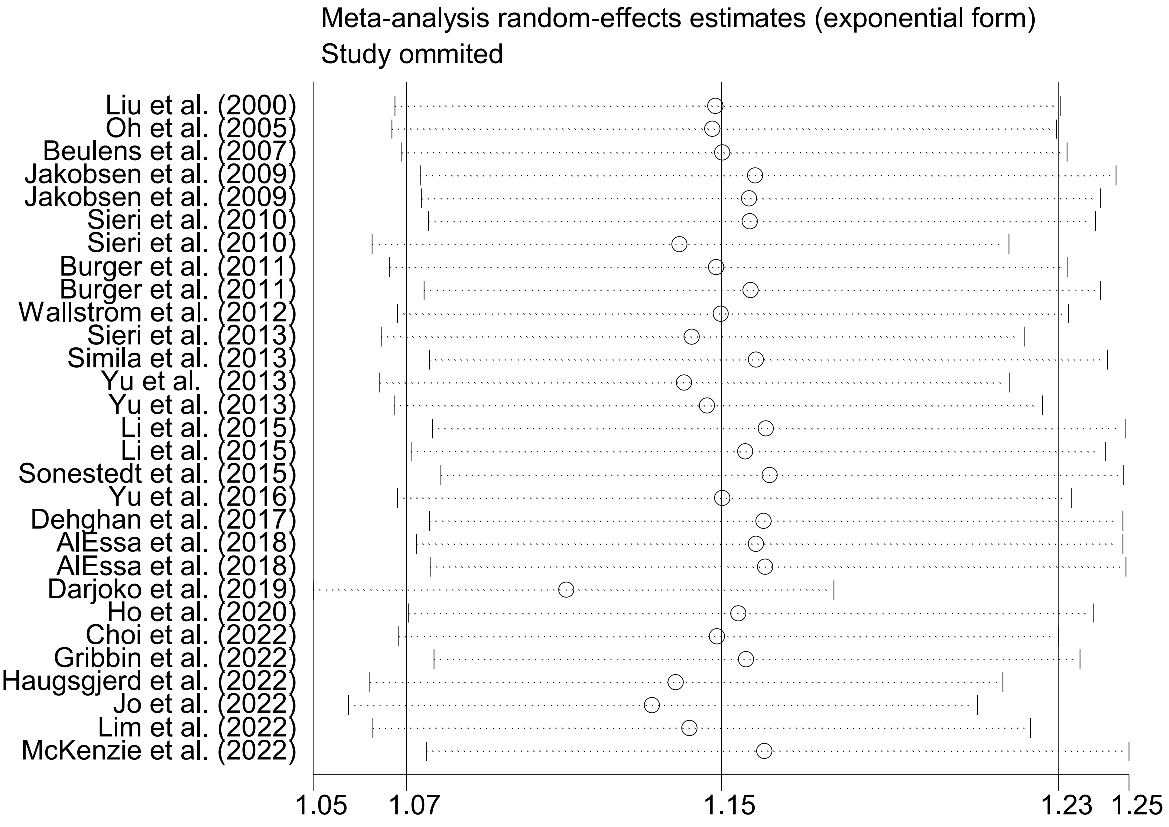

Figure S2 . Influence analysis of pooled hazard ratio/relative risks

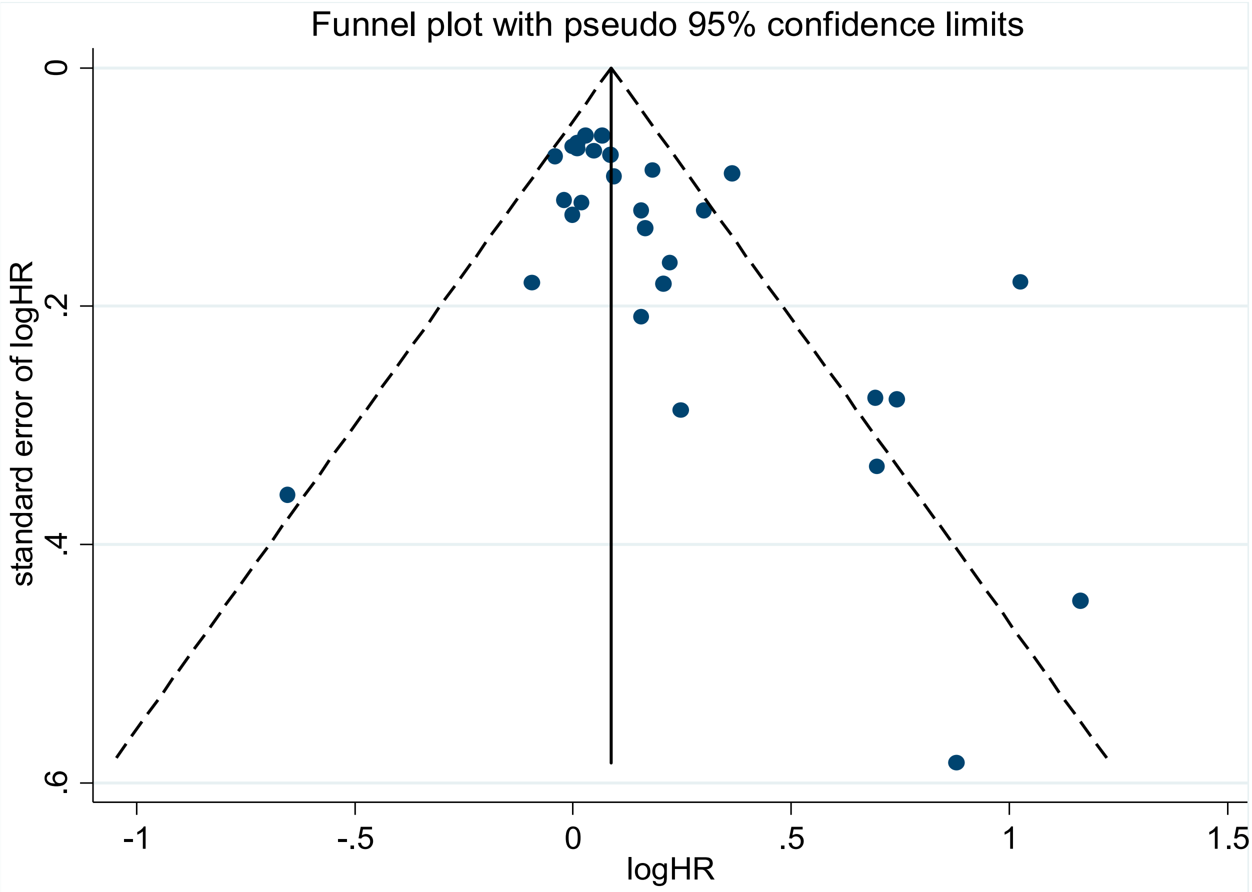

Figure S3. Funnel plot for publication bias

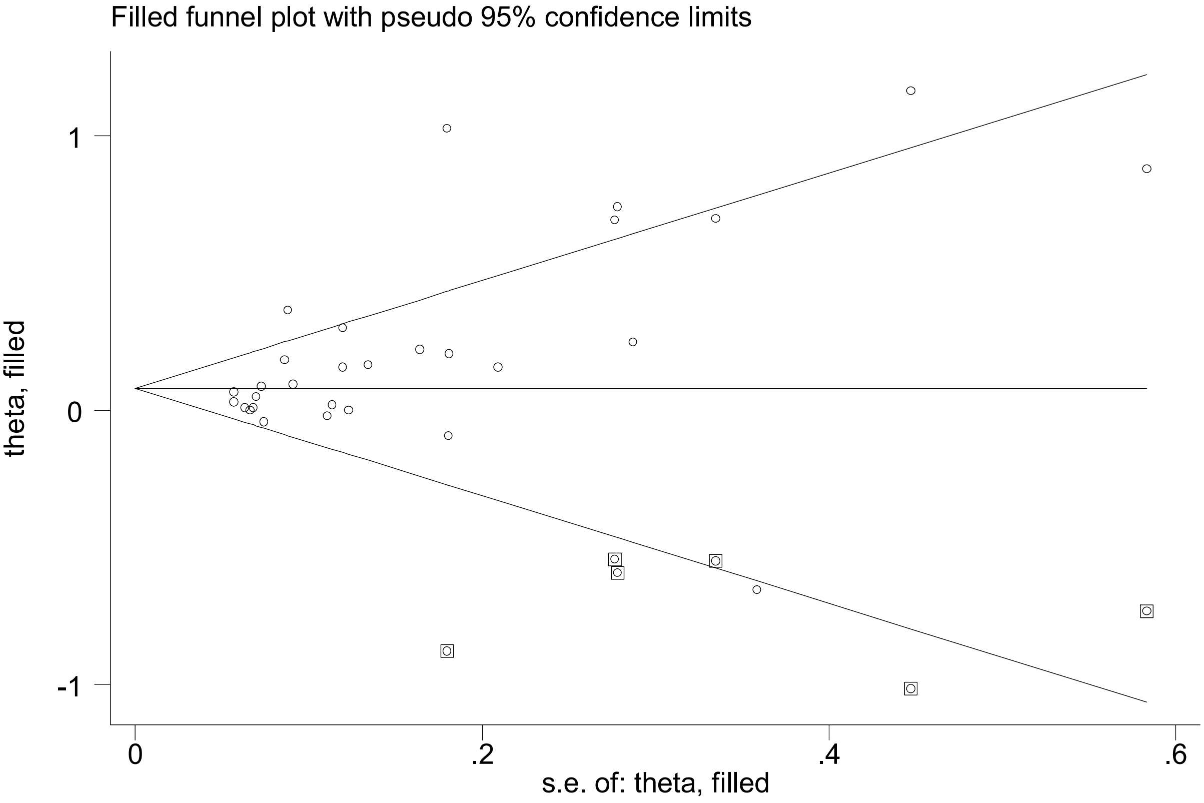

Figure S4. Trim and fill
